# Comparison of predictive performance for toxicity by accumulative dose of DVH parameter addition and DIR addition for cervical cancer patients

**DOI:** 10.1093/jrr/rraa099

**Published:** 2020-11-24

**Authors:** Yuya Miyasaka, Noriyuki Kadoya, Rei Umezawa, Yoshiki Takayama, Kengo Ito, Takaya Yamamoto, Shohei Tanaka, Suguru Dobashi, Ken Takeda, Kenji Nemoto, Takeo Iwai, Keiichi Jingu

**Affiliations:** Department of Radiation Oncology, Tohoku University Graduate School of Medicine, Sendai, Japan; Department of Heavy Particle Medical Science, Yamagata University Graduate School of Medical Science, Yamagata, Japan; Department of Radiation Oncology, Tohoku University Graduate School of Medicine, Sendai, Japan; Department of Radiation Oncology, Tohoku University Graduate School of Medicine, Sendai, Japan; Department of Radiation Oncology, Tohoku University Graduate School of Medicine, Sendai, Japan; Kanagawa Cancer Center, Yokohama, Japan; Department of Radiation Oncology, Tohoku University Graduate School of Medicine, Sendai, Japan; Department of Radiation Oncology, Tohoku University Graduate School of Medicine, Sendai, Japan; Department of Radiation Oncology, Tohoku University Graduate School of Medicine, Sendai, Japan; Course of Radiological Technology, Health Sciences, Tohoku University Graduate School of Medicine, Sendai, Japan; Course of Radiological Technology, Health Sciences, Tohoku University Graduate School of Medicine, Sendai, Japan; Department of Radiology, Yamagata University Faculty of Medicine, Yamagata, Japan; Department of Heavy Particle Medical Science, Yamagata University Graduate School of Medical Science, Yamagata, Japan; Department of Radiation Oncology, Tohoku University Graduate School of Medicine, Sendai, Japan

**Keywords:** cervical cancer, brachytherapy, deformable image registration, dose accumulation, GI toxicity

## Abstract

We compared predictive performance between dose volume histogram (DVH) parameter addition and deformable image registration (DIR) addition for gastrointestinal (GI) toxicity in cervical cancer patients. A total of 59 patients receiving brachytherapy and external beam radiotherapy were analyzed retrospectively. The accumulative dose was calculated by three methods: conventional DVH parameter addition, full DIR addition and partial DIR addition. ${D}_{2{cm}^3}$, ${D}_{1{cm}^3}$ and ${D}_{0.1{cm}^3}$ (minimum doses to the most exposed 2 cm^3^, 1cm^3^ and 0.1 cm^3^ of tissue, respectively) of the rectum and sigmoid were calculated by each method. V50, V60 and V70 Gy (volume irradiated over 50, 60 and 70 Gy, respectively) were calculated in full DIR addition. The DVH parameters were compared between toxicity (≥grade1) and non-toxicity groups. The area under the curve (AUC) of the receiver operating characteristic (ROC) curves were compared to evaluate the predictive performance of each method. The differences between toxicity and non-toxicity groups in ${D}_{2{cm}^3}$ were 0.2, 5.7 and 3.1 Gy for the DVH parameter addition, full DIR addition and partial DIR addition, respectively. The AUCs of ${D}_{2{cm}^3}$ were 0.51, 0.67 and 0.57 for DVH parameter addition, full DIR addition and partial DIR addition, respectively. In full DIR addition, the difference in dose between toxicity and non-toxicity was the largest and AUC was the highest. AUCs of V50, V60 and V70 Gy were 0.51, 0.63 and 0.62, respectively, and V60 and V70 were high values close to the value of ${D}_{2{cm}^3}$ of the full DIR addition. Our results suggested that the full DIR addition may have the potential to predict toxicity more accurately than the conventional DVH parameter addition, and that it could be more effective to accumulate to all pelvic irradiation by DIR.

## INTRODUCTION

Radiotherapy has played a crucial role in the treatment of gynecological malignancies, and the availability of clinical radiotherapy has been discussed for decades [1, 2]. For locally advanced cervical cancer, the combination of external beam radiotherapy (EBRT) and brachytherapy (BT) is the standard treatment. In recent years, 3D image-guided BT (3D-IGBT), using computed tomography (CT) and magnetic resonance imaging (MRI), has been widely employed worldwide and the 3D dose distribution and dose volume histogram (DVH) has been evaluated [3, 4].

To assess combination radiotherapy, the doses of EBRT and BT need to be accumulated. The Groupe Européen de Curiethérapie and the European SocieTy for Radiotherapy & Oncology (GEC-ESTRO) recommend calculating and reporting DVH parameters such as ${D}_{2{cm}^3}$, ${D}_{1{cm}^3}$ and ${D}_{0.1{cm}^3}$ (minimum doses to the most exposed 2 cm^3^, 1cm^3^ and 0.1 cm^3^ of tissue, respectively) to evaluate the dose irradiated to organs at risk (OAR). Frequently, simply adding DVH parameters of EBRT and each BT session is used to predict OAR toxicity. There is concern that conventional DVH parameter addition is based on the assumption that hotspots were located in the same regions as the OARs. However, under various clinical situations, inserting an applicator, shrinkage of the tumor and many other factors can cause changes in the relationships between organs. Therefore, high-dose regions on OARs are not always contiguous. Accordingly, the DVH parameter calculated by DVH parameter addition may overestimate the dose of OAR [4].

To solve this problem, there have been many attempts to use deformable image registration (DIR) to calculate the true accumulated dose. DIR enables the creation of accumulative dose distribution while taking into account the displacement and deformation of organs [5–12]. Andersen *et al*. reported that dose deviation of >5% between DVH parameter additions and DIR methods occurred in 2 and 38% of patients for ${D}_{2{cm}^3}$ and ${D}_{0.1{cm}^3}$ of the bladder, respectively [5]. Abe *et al*. evaluated rectal doses with DVH parameter and DIR additions and suggested that DVH parameter additions were overestimated by 3.1, 3.7 and 5.5 Gy for ${D}_{2{cm}^3}$, ${D}_{1{cm}^3}$ and ${D}_{0.1{cm}^3}$, respectively [6]. Furthermore, many other studies have reported the possible results when DVH parameter additions cause overestimation of the OAR dose [11, 12]. However, these studies have only compared DVH parameters between conventional DVH parameter additions and DIR additions, and the efficacy of using DIR for clinical outcomes has not been determined. Within this context, Zakariaee *et al*. retrospectively evaluated the relationship between DVH parameters calculated with DIR additions and bladder toxicity in patients with cervical cancer [13]. Kobayashi *et al*. revealed that DIR enabled EBRT and BT cumulative surface dose at the location of radiation injury [14]. In order to prove that DIR additions could take the place of conventional DVH parameter additions, it is necessary to directly compare the potential of both methods in predicting OAR morbidity. However, the superiority of DIR additions over conventional DVH parameter additions to predict clinical outcomes has not been shown.

For this purpose, we evaluated the correlation between gastrointestinal (GI) toxicity and DVH parameters calculated using accumulative dose and compared the predictive performance of toxicity between conventional DVH parameter addition and DIR addition.

**Table 1:** Patient characteristics^a^

Age, years	33–85 (median: 55)	
FIGO Stage		
IB	8	(14%)
IIA	2	(2%)
IIB	33	(56%)
IIIB	14	(24%)
IVA	1	(2%)
IVB	1	(2%)
Pathology		
SqCC	49	(83%)
Adeno	8	(14%)
Other	2	(3%)
Rectal toxicity		
Grade 0	42	(71%)
Grade 1	12	(20%)
Grade 2	3	(5%)
Grade 3	1	(2%)
Grade 4	1	(2%)
Chemotherapy		
+	48	(81%)
−	11	(19%)
Radiotherapy		
External beam irradiation		
Whole pelvic irradiation		
50.4Gy/28 fr	5	(8%)
30Gy/15 fr	36	(61%)
Other	8	(31%)
Central shielding irradiation		
20Gy/10 fr	39	(66%)
10Gy/5 fr	12	(20%)
Other	6	(10%)
Boost to lymph node		
10Gy/5 fr	11	(19%)
6Gy/3 fr	10	(17%)
Other	3	(8%)
Brachytherapy		
Tandem and ovoid	37	(63%)
Hybrid	22	(37%)

^a^FIGO = International Federation of Gynecology and Obstetrics, SqCC = squamous cell carcinoma, Adeno = adenocarcinoma, fr = fraction.

## MATERIALS AND METHODS

### Patient characteristics and treatment planning

This study was a retrospective single-institution analysis, and it was approved by our Institutional Review Board (2019–1-1002). Between 2015 and 2018, 59 patients, who were treated using a combination of EBRT and high-dose-rate brachytherapy (HDR-BT), were studied. A summary of patient characteristics, treatment planning and toxicity is shown in Table 1. The median follow-up time for all patients was 35 months (range, 7–60 months). All patients received whole pelvic (WP) irradiation EBRT. In addition, 57 patients underwent central shielding (CS) irradiation EBRT. The CS EBRT plan was created with the anterior–posterior/posterior–anterior (AP/PA) parallel-opposed field technique with a 4 cm midline block with a multi-leaf collimator. CS is used to avoid an overdose to the rectum and bladder, areas where the patient receives the highest HDR-BT dose. Furthermore, an EBRT boost to the lymph nodes was used to 24 patients. EBRT treatment planning dose calculations were performed using the Anisotropic Analytical Algorithm implemented in Eclipse version 11.0 (Varian Medical Systems, Palo Alto, USA). All patients received 2–5 treatments with ^192^Ir HDR-BT, usually once per week for consecutive weeks. Either a tandem and ovoid applicator or a hybrid BT, a tandem and ovoid applicator with implant plastic needles added [15], was used for BT. A total of 37 patients received tandem and ovoid applicators and 22 received a hybrid BT. After the applicator was inserted into the vagina, gauze was packed on the anterior and posterior sides of the applicator. In the treatment room, a CT image was acquired for planning using an Aquillion LB (Canon Medical Systems, Otawara, Japan). Contouring on CT images was performed using MRI as a reference. The treatment planning system used for HDR-BT was Oncentra version 4.1 (Elekta, Stockholm, Sweden), and TG-43 U1 methods were used for dose calculation [16]. The HDR-BT plan was optimized to prescribe the high-risk clinical target volume (HR-CTV) D90 in each HDR-BT session. The dose constraint was 75 Gy in the ${D}_{2{cm}^3}$ of the rectum and 90 Gy in the ${D}_{2{cm}^3}$ of the bladder. The CS EBRT and boost EBRT plans were not considered for the DVH parameter additions to calculate dose constraints because the irradiation fields of these plans did not completely overlap the region where the rectum and bladder received the highest BT dose. The DVH value was calculated during each treatment planning session and was approved by a physician prior to dose delivery.

### DIR

For calculation of accumulative dose distribution, DIR between each CT image was performed. The treatment planning CT image for the first fraction of BT and other EBRT and BT images (except for the first BT fraction) were used as a reference image and as moving images, respectively. As a preliminary step to DIR, the moving CT images were rigidly fused by matching the rectum and sigmoid structures with the reference image in a manual setting. After that, all moving images were deformed to the reference images using DIR. For the DIR algorithm, we used the ANAtomically CONstrained Deformation Algorithm (ANACONDA; implemented in RayStation ver. 6.2; RaySearch Laboratories, Stockholm, Sweden), which is considered to have hybrid intensity and structure-based DIR [17]. This DIR algorithm combines image information by intensity with the structure provided by the contours. In this study, we analyzed the toxicity of the rectum or sigmoid, which is a major concern in cervical cancer toxicity evaluation. Therefore, DIR was performed by setting rectum and sigmoid to the controlling region of interest (ROI). All other parameters were set to defaults. The Dice similarity coefficient (DSC) was calculated to evaluate DIR accuracy [18]. DSC is a common measure of the spatial overlap between contours [19], and it is defined with the following formula:

(1) \begin{equation*} \mathrm{DSC}=\frac{V_d\cap{V}_r}{\left({V}_d+{V}_r\right)\!\left/ \!2\right.} \end{equation*}

In this formula, ${V}_d$ represents the contours deformed by DIR, and ${V}_r$ represents the contours of the reference images. We also evaluated DIR accuracy using Hausdorff distance (HD) [20]. HD is defined as the maximum closest distance between two volume where the closest distance is computed for each vertex two volumes. HD is a measurement often used to evaluate DIR accuracy [21, 22].

### Creation of accumulative dose using DIR

**Fig. 1.**
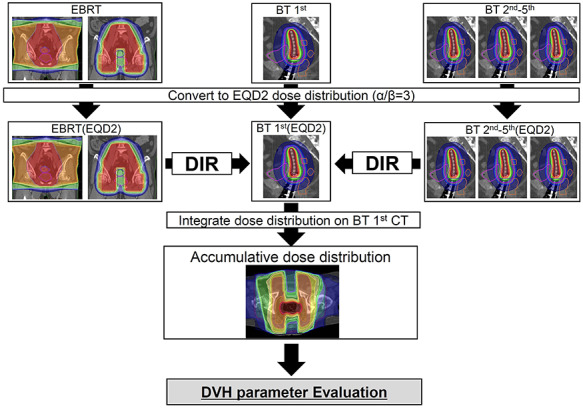
Schematic diagram of accumulating dose distribution with DIR. Each converted biologically equivalent dose in 2 Gy was deformed depending on DVH calculated by DIR between CT images. All deformed doses were integrated on BT first treatment planning CT.

The framework of creation of accumulative dose using DIR is shown in Fig. 1. Before calculating the accumulative dose, all EBRT and BT doses were converted to EQD2 on a voxel-by-voxel level using MATLAB 2017b (MathWorks, MA, USA) using linear-quadratic model-based equations with an $\alpha \!\Big/ \!\beta \Big.$ value of 3 Gy for late toxicity [23]. All dose distributions on the moving images were deformed on the basis of deformation vector fields calculated by DIR between each CT image shown in the previous section, and all deformed dose distributions were integrated on the reference image. In this study, we carried out full DIR addition and partial DIR addition. We calculated the full DIR addition by accumulation of all doses of WP, CS, boost to lymph nodes and BT using DIR. In contrast, we calculated the partial DIR addition by the accumulation of BT doses using the DIR and DVH parameter value of WP. Therefore, partial DIR addition does not involve CS doses and lymph node boost. We assumed that the CS of EBRT was made not to overlap with the BT dose distribution, and lymph node boost has little effect on the rectum and bladder as well, because CS and lymph node boost were originally planned based on the concept of not having a high dose overlap with BT. Therefore, in the partial DIR addition, even if EBRT included CS and lymph node boost, these were considered to have no overlap of high dose with BT, and only the WP of EBRT was added.

**Table 2:** The mean value DVH parameters for rectum + sigmoid of all patients

	${\boldsymbol{D}}_{\mathbf{2}{\boldsymbol{cm}}^{\mathbf{3}}}$		${\boldsymbol{D}}_{\mathbf{1}{\boldsymbol{cm}}^{\mathbf{3}}}$		${\boldsymbol{D}}_{\mathbf{0.1}{\boldsymbol{cm}}^{\mathbf{3}}}$	
	Mean ± SD	95% CI	Mean ± SD	95% CI	Mean ± SD	95% CI
DVH parameter addition	65.7 ± 8.8	(63.5–68.0)	70.7 ± 9.8	(68.1–73.2)	84.3 ± 13.7	(80.7–87.8)
Full DIR addition	68.9 ± 9.2	(66.6–71.3)	72.1 ± 10.5	(69.4–74.8)	81.5 ± 14.0	(77.8–85.1)
Partial DIR addition	61.6 ± 11.3	(58.7–64.6)	66.5 ± 10.8	(63.7–69.3)	75.9 ± 13.5	(72.4–79.4)
	**V50 Gy (cm** ^**3**^**)**		**V60 Gy (cm** ^**3**^**)**		**V70 Gy (cm** ^**3**^**)**	
	mean ± SD	95% CI	mean ± SD	95% CI	mean ± SD	95% CI
Full DIR addition	37.1 ± 27.2	(30.1–44.2)	15.7 ± 17.1	(11.2–20.2)	3.2 ± 4.5	(2.0–4.4)

### Comparison of DVH parameters and toxicity prediction performance in three methods

${D}_{2{cm}^3}$, ${D}_{1{cm}^3}$, ${D}_{0.1{cm}^3}$, V50, V60 and V70 Gy (volume irradiated over 50, 60 and 70 Gy, respectively) of rectum + sigmoid ROI were calculated. ${D}_{2{cm}^3}$, ${D}_{1{cm}^3}$ and ${D}_{0.1{cm}^3}$ were calculated by all three methods, while V50Gy, V60Gy and V70Gy, were calculated only by the full DIR addition because calculation of V50Gy, V60Gy and V70Gy requires the dose information in all voxels. It should be noted that all DVH parameters were calculated by the ROI of rectum + sigmoid, which is the sum of rectum and sigmoid. This is because the toxicity events of interest, especially intestinal bleeding, are considered to be a single event, regardless of whether they occur in rectum or sigmoid. The DVH parameters calculated by each accumulative method were compared among three accumulative methods and between toxicity groups, which were designated as either grade 1 or greater GI toxicity group (CTCAE ver. 4.0) or non-toxicity group. In this study, we evaluated the predictive performance of grade 1 or greater toxicity event assessment because there were fewer grade 2 or greater events in the analyzed patient group. In addition, for assessment of the predictive performance, the area under the curve (AUC) values in the receiver operating characteristic (ROC) curves for each accumulative method were compared [24]. We calculated the 95% confidence interval (CI) for AUC using bootstrap methods. We performed bootstrap methods with 1000 samples.

### Statistical analysis

We used the Wilcoxon rank sum test to compare DVH parameter values calculated by each method and between the toxicity and non-toxicity groups because normality was not confirmed using the Shapiro–Wilk test. The difference in AUC between each method was evaluated by chi-squared test. All statistical analyses were performed using JMP^®^ Pro Ver.14.1.0.

**Table 3:** Mean value DVH parameters for rectum + sigmoid of toxicity and non-toxicity group. A significant difference between toxicity and non-toxicity groups is indicated by ^*^P < 0.05

	${\boldsymbol{D}}_{\mathbf{2}{\boldsymbol{cm}}^{\mathbf{3}}}$					${\boldsymbol{D}}_{\mathbf{1}{\boldsymbol{cm}}^{\mathbf{3}}}$					${\boldsymbol{D}}_{\mathbf{0.1}{\boldsymbol{cm}}^{\mathbf{3}}}$				
	Toxicity (+)		Toxicity (−)			Toxicity (+)		Toxicity (−)			Toxicity (+)		Toxicity (−)		
	Mean ± SD	95% CI	Mean ± SD	95% CI	*P*-value	Mean ± SD	95% CI	Mean ± SD	95% CI	*P*-value	Mean ± SD	95% CI	Mean ± SD	95% CI	*P*-value
DVH parameter addition	65.9 ± 8.4	(61.5–70.9)	65.7 ± 9.0	(62.9–68.5)	0.94	71.1 ± 9.9	(66.0–76.2)	70.5 ± 9.8	(67.5–73.6)	0.80	85.5 ± 15.3	(77.6–93.4)	83.8 ± 13.2	(79.7–87.9)	0.76
Full DIR addition	73.0 ± 10.6	(67.6–78.5)	67.3 ± 13.3	(64.8–69.8)	^*^0.04	75.9 ± 12.7	(69.4–82.4)	70.6 ± 9.2	(67.7–73.5)	0.15	86.5 ± 16.3	(78.1–94.9)	79.4 ± 12.5	(75.5–83.3)	0.13
Partial DIR addition	63.8 ± 9.9	(58.8–68.9)	60.7 ± 11.9	(57.0–64.4)	0.41	68.1 ± 11.0	(62.5–73.7)	65.9 ± 10.8	(62.5–69.3)	0.52	78.1 ± 15.0	(70.4–85.8)	75.0 ± 12.9	(71.0–79.0)	0.54
	**V50 Gy (cm** ^**3**^ **)**					**V60 Gy (cm** ^**3**^ **)**					**V70 Gy (cm** ^**3**^ **)**				
	Toxicity (+)		Toxicity (−)			Toxicity (+)		Toxicity (−)			Toxicity (+)		Toxicity (−)		
	Mean ± SD	95% CI	Mean ± SD	95% CI	*P*-value	Mean ± SD	95% CI	Mean ± SD	95% CI	*P*-value	Mean ± SD	95% CI	Mean ± SD	95% CI	*P*-value
Full DIR addition	33.7 ± 17.8	(24.5–42.9)	38.5 ± 30.2	(29.1–47.9)	0.89	17.0 ± 11.5	(11.1–22.9)	15.2 ± 19.0	(9.2–21.1)	0.13	4.5 ± 5.5	(1.7–7.3)	2.7 ± 4.0	(1.4–3.9)	0.08

## RESULTS

Regarding DIR accuracy, DSCs were 0.83 ± 0.15 and 0.72 ± 0.20 and the HDs were 18.2 ± 12.7 and 22.1 ± 12.8 mm for rectum and sigmoid, respectively.

Table 2 shows the mean ${D}_{2{cm}^3}$, ${D}_{1{cm}^3}$, ${D}_{0.1{cm}^3}$, V50Gy, V60Gy and V70Gy of all cases by each accumulative method. The mean ${D}_{2{cm}^3}$, ${D}_{1{cm}^3}$ and ${D}_{0.1{cm}^3}$ calculated by DVH parameter addition were significantly higher than that of partial DIR addition for all parameters (*P* < 0.05). Regarding the full DIR addition, ${D}_{2{cm}^3}$, ${D}_{1{cm}^3}$ and ${D}_{0.1{cm}^3}$ were significantly higher than the partial DIR addition (*P* < 0.05).

Table 3 shows the summary of ${D}_{2{cm}^3}$, ${D}_{1{cm}^3}$, ${D}_{0.1{cm}^3}$, V50Gy, V60Gy and V70Gy in toxicity and non-toxicity groups. The results of the DVH parameter addition showed that the dose differences between the two groups were 0.2, 0.6 and 1.7 Gy for ${D}_{2{cm}^3}$, ${D}_{1{cm}^3}$ and ${D}_{0.1{cm}^3}$, respectively, and no significant differences were observed between the groups for any parameter. In contrast, in the full DIR addition, the ${D}_{2{cm}^3}$, ${D}_{1{cm}^3}$ and ${D}_{0.1{cm}^3}$ of toxicity groups were 5.7, 5.3 and 7.1 Gy higher than the non-toxicity group, respectively. There was a significant difference in dose between the two groups in ${D}_{2{cm}^3}$ (*P* = 0.04). The dose difference between ${D}_{2{cm}^3}$, ${D}_{1{cm}^3}$ and ${D}_{0.1{cm}^3}$ in the partial DIR addition between the toxicity and non-toxicity groups was 3.1, 2.2 and 3.1 Gy, and the dose tended to be higher in the toxicity group. For V50Gy, V60Gy and V70Gy, the differences between the toxicity and non-toxicity groups were 4.8, 1.8 and 1.8 cm^3^, respectively, and were higher in the toxicity group than in the non-toxicity group in V60y and V70 Gy.

**Fig. 2.**
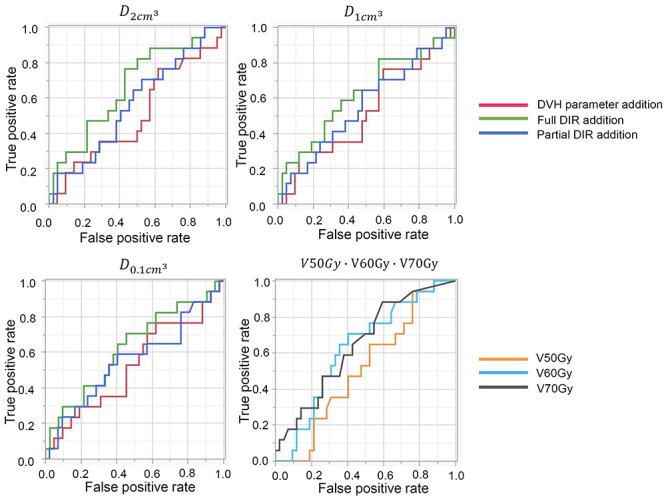
Comparison of ROC curves using each accumulative method. ROC shows the predicting performance of grade 1 or higher GI toxicity.

The results of the AUC are presented in Table 4 and Fig. 2. AUCs with ${D}_{2{cm}^3}$ in DVH parameter addition, full DIR addition and partial DIR addition were 0.51 (95% CI, 0.46–0.59), 0.67 (95% CI, 0.51–0.80) and 0.57 (95% CI, 0.48–0.70), respectively, and the full DVH parameter addition showed the highest AUC among three methods. Similar results were observed in ${D}_{1{cm}^3}$ and ${D}_{0.1{cm}^3}$. The AUC was 0.51 (95% CI, 0.39–0.60), 0.63 (95% CI, 0.39–0.77), and 0.65 (95% CI, 0.41–0.77) for V50y, V60y and V70 Gy, respectively, indicating a higher AUC for the parameters evaluated for higher dose regions such as V60Gy and V70Gy.

**Table 4:** AUC of predictive performance detecting ≥grade1 GI toxicity. A significant difference compared with DVH parameter addition is indicated by ^*^P < 0.05

	${\boldsymbol{D}}_{\mathbf{2}{\boldsymbol{cm}}^{\mathbf{3}}}$		*P* value (vs DVH parameter addition)	${\boldsymbol{D}}_{\mathbf{1}{\boldsymbol{cm}}^{\mathbf{3}}}$		*P* value (vs DVH parameter addition)	${\boldsymbol{D}}_{\mathbf{0.1}{\boldsymbol{cm}}^{\mathbf{3}}}$		*P* value (vs DVH parameter addition)
	AUC	95%CI		AUC	95%CI		AUC	95%CI	
DVH parameter addition	0.51	(0.46–0.59)		0.52	(0.46–0.58)		0.53	(0.45–0.60)	
Full DIR addition	0.67	(0.51–0.80)	< 0.01^*^	0.62	(0.49–0.77)	0.05^*^	0.63	(0.49–0.77)	0.03^*^
Partial DIR addition	0.57	(0.48–0.70)	0.06	0.55	(0.48–0.68)	0.37	0.55	(0.46–0.67)	0.58
	**V50 Gy (cm** ^**3**^**)**			**V60 Gy (cm** ^**3**^**)**			**V70 Gy (cm** ^**3**^**)**		
	AUC	95%CI		AUC	95%CI		AUC	95%CI	
Full DIR addition	0.51	(0.39–0.60)		0.63	(0.39–0.77)		0.65	(0.41–0.77)	

Among the analyzed cases, we observed a grade 4 toxicity in one case. ${D}_{2{cm}^3}$ of the case was 79.7, 99.0 and 81.9 Gy by DVH parameter addition, full DIR addition and partial DIR addition, respectively. The full DIR addition recorded the highest value among the three methods. The V50Gy, V60Gy and V70Gy values obtained by the full DIR addition were 55.4, 33.1 and 18.2 cm^3^, respectively. These values were respectively 18.3, 17.4 and 15.0 cm^3^ higher than the mean of all cases for each parameter.

## DISCUSSION

In the present study, to evaluate the clinical utility of DIR-based dose accumulation, we compared predictive performance between DVH parameter addition and DIR addition for patients with cervical cancer.

The full DIR addition had greater dose differences of ${D}_{2{cm}^3}$, ${D}_{1{cm}^3}$ and ${D}_{0.1{cm}^3}$, between the toxicity and non-toxicity groups than the other accumulative methods. In addition, the AUC of full DIR addition had the highest value among the methods examined in this study. These results suggest that the full DIR addition can predict toxicity events more accurately than other methods. Zakariaee *et al*. reported the relationship between bladder toxicity events and the accumulative dose using DIR, with parameters such as ${EQD}_{2{cm}^3}$ and ${EQD}_{1{cm}^3}$ being higher in the toxicity group than in the non-toxicity group [13]. This trend was similar in our study, and in the full DIR addition, the DVH parameter values were higher in the toxicity group. V50Gy, V60Gy and V70Gy are parameters that can be calculated using the full DIR addition. Of these, V60Gy and V70Gy were higher in the toxicity group, although there was no significant difference, and the AUC values of V60Gy and V70Gy were higher than any value calculated by the DVH parameter addition or partial DIR addition. Zakariaee *et al*. showed that volume-assessed parameters such as V3Gy and V5Gy may be useful for the assessment of bladder toxicity events in parameters calculated using DIR [13]. These results suggest that DVH parameters evaluated by volume using DIR may be useful in toxicity event assessment as well as in GI assessed in this study.

The difference in the toxicity prediction accuracy described above was expected to come from the difference in the accumulative dose calculated by each method. Several previous studies have shown that DVH parameter additions tended to be overestimated. Our results are consistent with their results (Table 2). These results indicated that this overestimation in DVH parameter addition may reduce the toxicity prediction accuracy. In addition, our result showed that the full DIR addition was higher than the partial DIR addition for all DVH parameters. This result is consistent with the result reported by Teo *et al*. [7]. It should be noted that the dose difference in our study between the full and partial DIR additions was larger than that in their study (e.g. dose difference in ${D}_{2{cm}^3}$,: 7.3 vs 2.0 Gy). There are two possible reasons for this. First, our study had a higher percentage of patients treated with CS with a heterogeneous dose distribution (96 vs 5%). Second, only our study evaluated the dose to sigmoid in addition to rectum for the evaluation of toxicity events. Considering these two reasons, it is likely that the tortuous sigmoid unexpectedly penetrated into the overlap region of CS and BT. If this is the case, more reasonable evaluation may be possible by using DIR when a heterogeneous dose distribution is used.

In this study, the AUC of full DIR addition was higher in the order of ${D}_{2{cm}^3}$, V70 Gy, V60 Gy, ${D}_{1{cm}^3}$ and ${D}_{0.1{cm}^3}$, The reason why AUCs of ${D}_{1{cm}^3}$ and ${D}_{0.1{cm}^3}$ were lower than that of ${D}_{2{cm}^3}$ may be that the smaller the volume to be evaluated the more susceptible it is to the DIR accuracy. Reniers *et al*. mentioned that even an error of ~2 mm could result in a dose error of about 5 ~ 10% [25]. Kadoya *et al*. reported that the difference between the accumulative doses using DIR with different accuracy was larger for ${D}_{0.1{cm}^3}$ than for ${D}_{2{cm}^3}$ [8] Therefore, toxicity prediction accuracy using the smaller volumes had stronger influence on DIR error. V60Gy and V70Gy may predict toxicity events as accurately as ${D}_{2{cm}^3}$. This may be due to the fact that V60Gy and V70Gy evaluate the same high dose area as ${D}_{2{cm}^3}$. The reason seems to be that the same high dose area as assessed by ${D}_{2{cm}^3}$ is associated with GI toxicity.

There were several limitations in this study. The first is the small number of cases and the fact that this was a single-center study. An increase in the number of cases and a multicenter study may yield more detailed and definitive results. In addition, the analysis of grade 1 or higher cases in this study may have resulted in some uncertainty. In the cases analyzed in this study, there were few cases of grade 2 or higher toxicity, which seems to be less uncertain in the determination of toxicity events. This may be due to compliance with OAR dose constraints during treatment planning. Further detailed results could be obtained by analyzing a group of patients with more grade 2 or higher toxicity cases. This study was limited to the evaluation of toxicity by DVH parameters, which did not involve the relationship between other clinical factors (age, presence or absence of chemotherapy) and toxicity. Although the dose proves to be an important factor in the occurrence of toxicity, other clinical factors have a significant influence as well. For example, a report suggested that grade 3 or higher intestinal toxicity is more likely to occur in cases with concurrent chemo-radiotherapy [26]. In addition, other reports postulate that age could be a factor influencing the occurrence of toxicity [27]. Sturdza *et al*. suggested that the use of bevacizmab may lead to a more severe toxicity [28], but our study could not evaluate the toxic effect of drugs. Combining DVH parameters deduced by DIR additions with clinical information may lead to further improvements in the accuracy of toxicity prediction. Another limitation was the DIR accuracy. Although we used the hybrid DIR implemented in RayStation, in which we got reasonable DIR accuracy in a previous study [29], the DIR had a residual error. We did additional analysis using the patients who had reasonable DIR accuracy (DSC > 0.8) and got similar result to results with all patients (Tables S1 and S2, see online supplementary material). Rigaud *et al*. evaluated the accuracy of DIR for sigmoid [30]. Results performed with ANACONDA (similar to our study), were 0.57 mm for DSC and 29.4 mm for HD, with a poorer accuracy compared to our results (e.g. DSC = 0.72 mm, HD = 22.1 mm). This is because the study primarily used image intensity as combined information, whereas we used ROI with image intensity as combined information. The study reported that a biomechanical model (MOLFEUS) and point set deformable algorithm (sTPS-RPM) can improve the DIR accuracy even with the sigmoid. Using such an accurate DIR algorithm will improve the accuracy of the accumulative dose, resulting to an improvement in the ability to predict adverse events. Other factors that give uncertainty in the accumulative dose is inter-fractional or intra-fractional error during treatment. van Heerden et al. evaluated the effect of intra-fractional motion on dose during EBRT in cervical cancer [31]. They showed that inter-fractional motion has a small effect on dose. In addition, although brachytherapy dose not consider the effect of intra-fractional motion during treatment [32–34], many studies have considered that the average errors were not large. Therefore, the effect of error during treatment, which is basically not evaluated in this study, is considered to be small. However, regarding the maximum error, van Heerden reported an error of up to 2.8 Gy [31], additionally D_2cm^3^_ showed an error of 1–6 Gy [32–34] in the evaluation of intra-fractional motion of brachytherapy. Pretreatment of gas and fecal can reduce these errors and, as a result, further improve the accumulative dose toxicity event prediction accuracy.

## CONCLUSION

In this study, we compared the predictive performance of GI toxicity between DIR addition and conventional DVH parameter addition. Our results indicated that full DIR addition may have the potential to predict toxicity with higher accuracy than DVH parameter addition. In addition, it was also found that the accumulative dose using DIR could be more effective by accumulating not only BT but also all pelvic irradiation including CS or other boost using DIR.

## CONFLICT OF INTEREST

There is no conflict of interest with regard to this manuscript.

## Supplementary Material

supplementary_data_CleanCopy_rraa099

TableS1_rraa099

TableS2_rraa099
