# Supplementary material for: Comparison of predictive performance for toxicity by accumulative dose of DVH parameter addition and DIR addition for cervical cancer patients
Source: J Radiat Res. 2020 Nov 24;62(1):155–62. doi: 10.1093/jrr/rraa099 (PMC7779363; doi:10.1093/jrr/rraa099)
Supplement: supplementary_data_CleanCopy_rraa099 [file supplementary_data_cleancopy_rraa099.docx]

**Supplementary data**

The results of an additional study focusing on cases with a mean DSC > 0.8 in rectum and sigmoid are shown below. There were 17 cases, 8 in the Toxicity group and 9 in the Non-toxicity group. Each DVH parameter in these cases is shown in Table S1. In addition, AUC values are given in Table S2.

On analyzing full DIR additions, there was no significant difference in the doses between toxicity and non-toxicity groups. This could be explained by a decrease in the number of cases limiting them to those with high DIR accuracy (DSC > 0.8). Although we had 59 cases analyzed in the main data, only 17 cases were analyzed in the supplementary data. This decrease in sample size led to an extension of the 95% confidence interval range. Moreover, this decrease led to the variation in the DVH parameter values rendering their comparison between toxicity and non-toxicity groups erroneous.


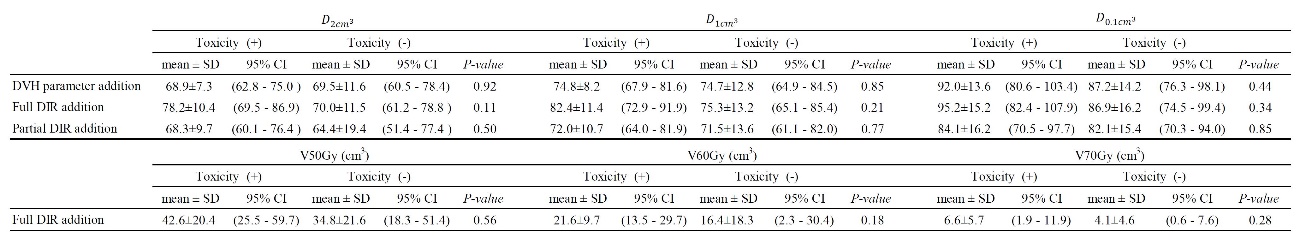


Table S1. The mean value of $D_{{2 cm}^{3}}$, $D_{{1 cm}^{3}}$, $D_{{0.1 cm}^{3}}$, V50Gy, V60Gy and V70Gy for rectum + sigmoid with DVH parameter addition and DIR addition between toxicity and non-toxicity group

Abbreviations: $D_{{2cm}^{3}}$ = minimum doses to the most exposed 2 cm^3^ of tissue, $D_{{1cm}^{3}}$ = minimum doses to the most exposed 1 cm^3^ of tissue, $D_{{0.1cm}^{3}}$ = minimum doses to the most exposed 0.1 cm^3^ of tissue, DVH = dose-volume histogram, DIR = deformable image registration. V50Gy = volume irradiated over 50 Gy, V60Gy = volume irradiated over 60 Gy, V70Gy = volume irradiated over 70 Gy, , 95% CI = 95% confidence interval


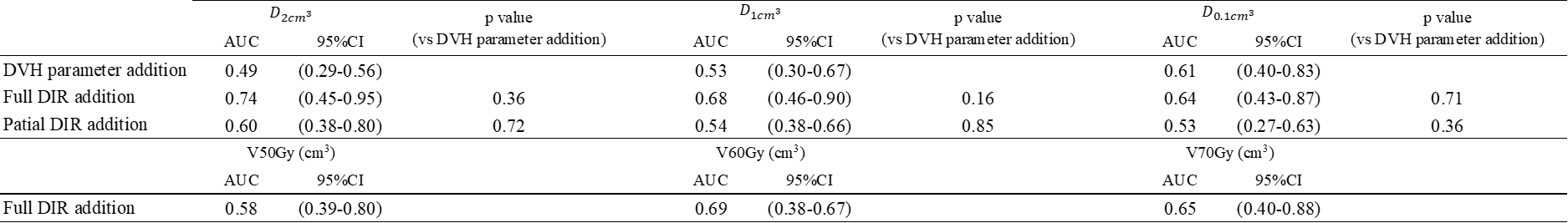


Table S2. AUC of predictive performance detecting ≥Grade1 rectal + sigmoid toxicity.

Abbreviations: AUC = area under the curve, $D_{{2cm}^{3}}$ = minimum doses to the most exposed 2 cm^3^ of tissue, $D_{{1cm}^{3}}$ = minimum doses to the most exposed 1 cm^3^ of tissue, $D_{{0.1cm}^{3}}$ = minimum doses to the most exposed 0.1 cm^3^ of tissue, DVH = dose-volume histogram, DIR = deformable image registration. V50Gy = volume irradiated over 50 Gy, V60Gy = volume irradiated over 60 Gy, V70Gy = volume irradiated over 70 Gy, , 95% CI = 95% confidence interval
