# Supplementary figures and images for: Comparison of predictive performance for toxicity by accumulative dose of DVH parameter addition and DIR addition for cervical cancer patients
Source: J Radiat Res. 2020 Nov 24;62(1):155–62. doi: 10.1093/jrr/rraa099 (PMC7779363; doi:10.1093/jrr/rraa099)

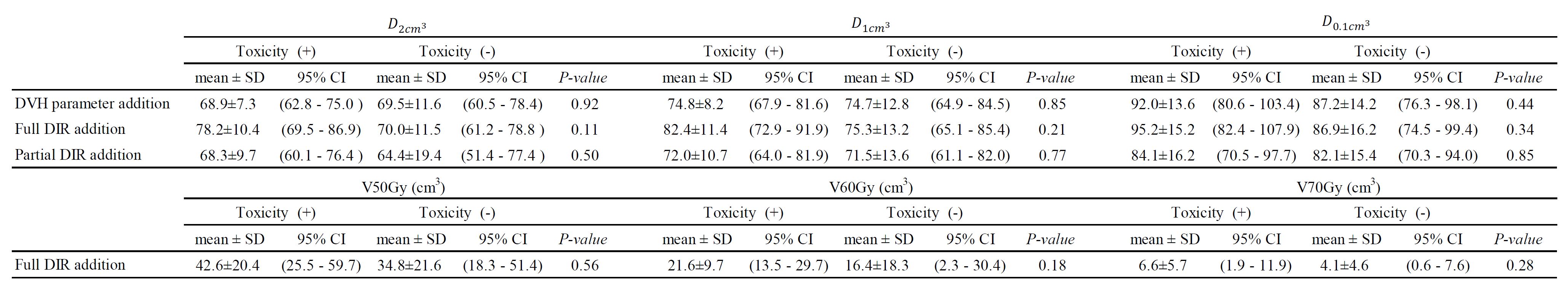

Supplement: TableS1_rraa099 [file tables1_rraa099.jpeg]

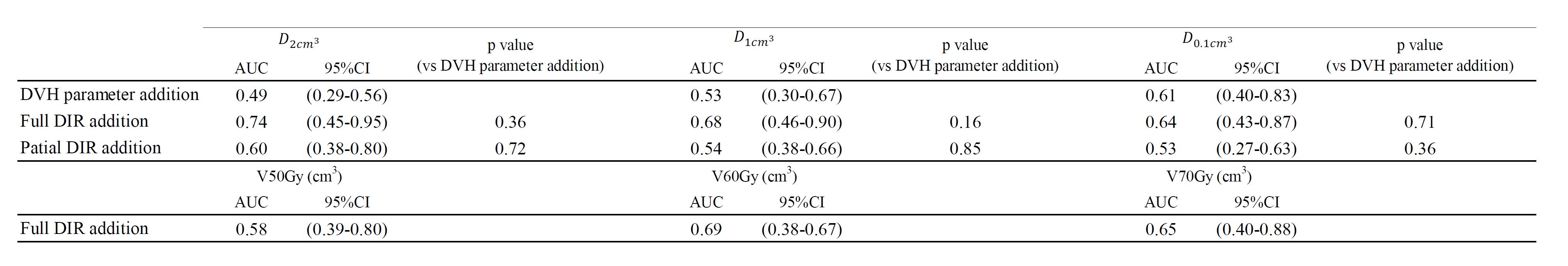

Supplement: TableS2_rraa099 [file tables2_rraa099.jpeg]
